# The Voluntary Hospitals of Manchester

**Published:** 1924-01

**Authors:** 


### THE VOLUNTARY HOSPITALS OF MANCHESTER.

### COMMITTEE'S REPORT.

IN July, 1921, the City Council of Manchester was requested by the Voluntary Hospitals' Commission to set up a Local Voluntary Hospital Committee. This Committee, under the Chairmanship of Mr. Walter Davies, has now issued its Report, a document of much interest, which, we note, may become an annual publication. The Committee was composed of two medical practitioners nominated jointly by the local medical committees for the area recognised by the Minister of Health, one in general practice and one on the staff of a voluntary hospital; two hospital representatives not medical practitioners, one representing the larger general hospitals and the other the smaller and cottage hospitals in the area; two members nominated by the City Council and five members, one at least having to be a woman, nominated by the Commissioners.

### Functions of the Committee.

The functions of the Committee were to be :—

To act as local advisers to the Commission; to collect information as to the needs of their areas; to further co-operation between hospitals; to co-ordinate appeals; to consider the possibility of arranging the transfer of patients where this can advantageously be done; to prepare, where practicable, schemes of co-operative purchase; to advise as to the adoption of a uniform system of hospital accounts throughout their area; to organise systematic contributions, both from employers and employees, in areas where no such systems at present exist; to undertake the distribution of any contributions made by an Approved Society in cases where the Society is purely local in character; and generally to take every possible step to assist the hospitals in their area to maintain the present voluntary system. They would also consider generally the existing provision of hospital accommodation in their area, and advise the Commission thereon.

### Combined Action.

The Local Committee have advised the Voluntary Hospitals' Commission as to the distribution of a sum amounting to £17,485 among the necessitous hospitals within their area, and £12,485 has already been paid over to the hospitals :—

The Committee have also been instrumental in the arrangement of a scheme for combined action by the Manchester and Salford Hospital Saturday and Convalescent Homes Fund and the Manchester and District Hospitals for raising funds from employers and employees. This scheme has been in operation for some time, and promises to give results of great financial advantage by the systematic collection of funds, thereby ensuring a steady annual income for the work of the voluntary hospitals.

### Some Statistics.

The total ordinary income for the year under review was £231,852, of which only 16 per cent. is subscribed voluntarily by patients; £38,997 is received from local authorities and approved societies; £51,476 from invested property, and the balance of £104,400 has to be met from various voluntary sources. The ordinary expenditure on maintenance is £281,276, and there is thus a deficiency for the year of £49,424. This, of course, does not take into account extraordinary income, which would still leave a deficit of over £15,000. Of the expenditure in maintenance the heaviest items are wages £94,493, provisions £60,118, and domestic £55,448. With respect to free legacies the Local Voluntary Hospitals Committee are of the opinion that as a matter of policy these should be invested in order to provide a regular source of income. Many of the hospitals have been unable to follow this course, and in order to meet the maintenance expenditure they have had to use legacies for current requirements. Out of an average number of 1,746 beds in daily occupation only 162 bring in any definite payment. The number of out-patients' attendances reaches the enormous total of 772,034, the number of in-patients admitted during the year being 27,787.

### Is the Voluntary System to Continue?

The Committee add that they are greatly impressed by the statistics as indicating the immense amount of work done to alleviate suffering. They continue :—

When it is realised that the services rendered are carried on with an ever-growing deficiency, the Committee feel that the public will rally to the help of the hospitals in a way which should enable them to proceed with and further extend their work, not only on the curative side, but also on the side of research. The Committee, having the foregoing facts in view, are also constrained to ask the question: "Is the Voluntary System worth continuing?" They hold the same views on the matter as were expressed by the Cave Committee, but it appears to the Local Voluntary Hospitals Committee that it is for the public themselves to answer the question. If the answer is in the affirmative, then greater support will have to be forthcoming. If the answer is in the negative, and this will only be ascertained by the non-support of the hospitals, most surely the institutions will become either State or Municipal institutions, with all the objections to which such institutions are liable.

